# Perinatal Vitamin D Deficiency Enhances Brown Adipose Tissue Thermogenesis in Weanling Rats

**DOI:** 10.3390/ijms26104534

**Published:** 2025-05-09

**Authors:** Matheus L. Moro, Natany G. Reis, Aline Z. Schavinski, João B. Camargo Neto, Ana Paula Assis, Jonathas R. Santos, Luciane C. Albericci, Isis C. Kettelhut, Luiz C. C. Navegantes

**Affiliations:** 1 Department of Physiology, Ribeirão Preto Medical School, University of São Paulo, Ribeirão Preto 14049-900, SP, Brazil; matheusmoro@usp.br (M.L.M.); natany.garcia.reis@gmail.com (N.G.R.); alinezanatta@usp.br (A.Z.S.); 2 Department of Biochemistry/Immunology, Ribeirão Preto Medical School, University of São Paulo, Ribeirão Preto 14049-900, SP, Brazil; batistajoao@usp.br (J.B.C.N.); assis.anap92@gmail.com (A.P.A.); idckette@fmrp.usp.br (I.C.K.); 3 Department of BioMolecular Sciences, Faculty of Pharmaceutical Sciences of Ribeirão Preto, University of São Paulo, Ribeirão Preto 14040-900, SP, Brazil; jonathas.rodrigo.santos@usp.br (J.R.S.); alberici@fcfrp.usp.br (L.C.A.)

**Keywords:** vitamin D, perinatal vitamin D deficiency, brown adipose tissue, thermogenesis, fetal programming

## Abstract

Perinatal vitamin D (Vit. D) deficiency (VDD) disrupts the development of key tissues involved in metabolic regulation, including the endocrine pancreas, white adipose tissue, and skeletal muscle. Brown adipose tissue (BAT), essential for thermoregulation and energy homeostasis, may also be affected, but the impact of perinatal VDD on BAT physiology remains unclear. In this study, forty female Wistar rats were fed either a standard AIN93G diet (1000 IU Vit. D_3_/kg; control group, CT) (*n* = 20) or a modified AIN93G diet lacking Vit. D (VDD group) (*n* = 20) for six weeks prior to conception and throughout gestation and lactation. Male offspring were evaluated at weaning (PN21) and adulthood (PN180) after Vit. D status was normalized through a standard diet. We found that perinatal VDD reduced total lipid droplet area, increased oxygen consumption, and upregulated thermogenic gene expression in BAT at weaning. Correspondingly, VDD offspring exhibited greater cold tolerance and enhanced BAT recruitment upon cold exposure (4 °C). Notably, normalization of Vit. D status by adulthood fully reversed these changes, indicating that while perinatal VDD transiently enhances BAT thermogenic activity during early life, it does not produce lasting effects into adulthood.

## 1. Introduction

Vit. D is a steroid hormone primarily involved in bone mineral homeostasis [1]. It can be obtained from the diet as cholecalciferol (Vit. D3) or ergocalciferol (Vit. D2) and synthesized in the skin after sun exposure, particularly in humans [2]. Both forms are metabolized into 25(OH)D3 (calcidiol) in the liver [3]. Calcidiol is the long-lasting inactive form in the bloodstream, serving as a biomarker of whole-body Vit. D status [4]. In the kidneys, CYP27B1 converts calcidiol into 1,25(OH)_2_D3 (calcitriol), the most active metabolite [5]. Calcitriol exerts its effects mainly through the nuclear Vit. D receptor (VDR), regulating over 300 target genes [6,7]. Besides its well-established role in enhancing calcium absorption, suppressing parathyroid hormone (PTH) secretion, and promoting bone turnover [8,9,10], calcitriol also exerts immunomodulatory [11] and antiproliferative effects [12], stimulates insulin secretion [13], and inhibits the renin–angiotensin system [14]. Consequently, Vit. D plays a pivotal role in human health, with VDD being associated with conditions such as rickets, osteomalacia [15], respiratory tract infection [16], Type 1 Diabetes Mellitus [17], and hypertension [18].

The Developmental Origins of Health and Disease (DOHaD) concept suggests that nutritional imbalance during perinatal life may trigger adaptive responses in offspring [19]. A recent meta-analysis showed that VDD is a common health issue in women of childbearing age [20]. Animal and clinical studies link maternal VDD during perinatal life to negative outcomes in the maternal–fetal dyad [21], including gestational diabetes [22], low birth weight [23], and neurological disorders [24]. Our group demonstrated that maternal VDD during gestation and lactation induces sex-specific atrophy of type-II muscle fibers in newly weaned male Wistar rats. This phenotype is followed by compensatory changes in adulthood, such as increased muscle calcitriol content, hypertrophy, and higher resistance to fatigue [25]. We also showed that perinatal VDD reduces pancreatic β-cell mass and impairs in vitro insulin secretion in adult male offspring [26]. Thus, Vit. D may play a broader role in regulating the development and function of tissues involved in metabolic control.

Brown adipose tissue (BAT) consists of multilocular adipocytes rich in mitochondria that dissipate energy as heat through non-shivering thermogenesis [27]. This process is mediated by the expression of uncoupling protein 1 (UCP-1) in the mitochondrial membrane [28,29]. UCP-1 activity is stimulated by intracellular fatty acids, uncoupling oxidative phosphorylation from ATP synthesis [30]. Once activated, BAT takes up metabolic substrates from the blood and increases energy expenditure [31,32,33]. Until 2009, BAT was thought to exist only in newborns, where it played a key role in maintaining body temperature independently of shivering [34]. PET-CT techniques later identified metabolically active BAT in adult humans [35,36,37,38]. Moreover, observational studies have since linked higher BAT activity to lower risks of type 2 diabetes, cardiovascular disease, and central obesity [39,40], driving greater interest in its development and regulation.

In rodents, brown adipocytes develop at embryonic day 15.5 from Myf-5+ mesenchymal progenitors that also give rise to skeletal muscle [41]. Prdm16 is the key transcription factor that suppresses the myogenic program and induces thermogenic genes in these cells [42]. Once differentiated, BAT activity is controlled by the sympathetic nervous system (SNS) through norepinephrine (NOR) release, which activates non-shivering thermogenesis by stimulating lipolysis and inducing thermogenic genes [43,44]. In addition to SNS control, several nutritional and hormonal factors regulate BAT function. Studies show that Vit. D suppresses brown adipocytes differentiation and respiratory capacity [45,46], even though its role in BAT development remains unclear.

Therefore, we hypothesize that diminished Vit. D signaling, induced by a perinatal nutritional deficiency model, augments BAT thermogenic function in weanling rats and that it could permanently program the tissue during adult life. Our findings demonstrated that perinatal VDD activates the BAT thermogenic program at weaning independently of hypocalcemia, but this effect reversed in adult life after the recovery of Vit. D nutritional status.

## 2. Results

### 2.1. Maternal Vit. D Deficiency During Gestation and Lactation Affects Perinatal Vit. D Status in the Offspring

**Figure 1:**
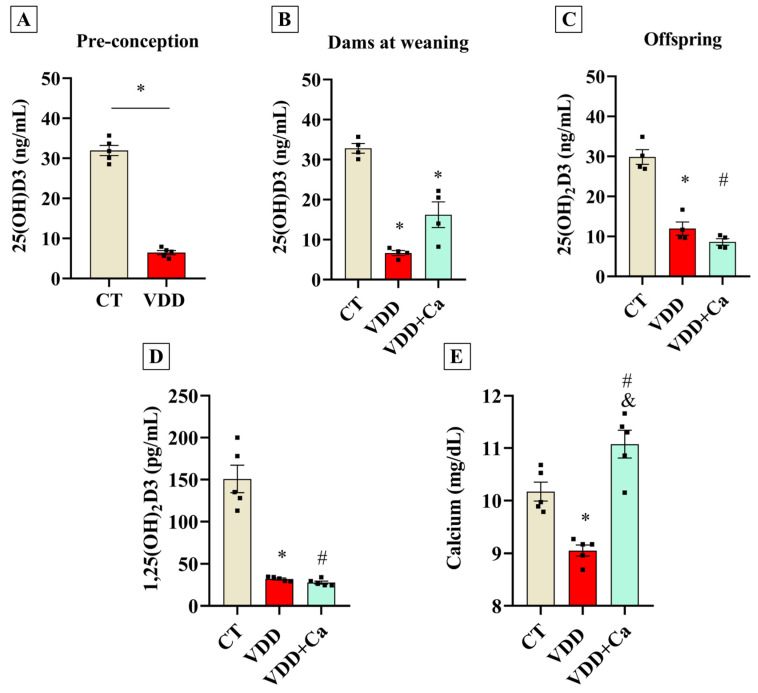
Vit. D−related metabolites in serum of Vit. D−sufficient (CT), Vit. D−deficient (VDD), and Vit. D−deficient supplemented with calcium (VDD + Ca) dams and their offspring. (**A**) Serum 25OHD3 (calcidiol) concentrations in CT and VDD dams in pre−conceptional period (*n* = 5). (**B**) Serum 25OHD3 (calcidiol) concentrations in CT, VDD, and VDD + Ca dams at weaning (PN21) (*n* = 4). (**C**) Serum 25OHD3 (calcidiol) concentration. (**D**) Serum 1,25(OH)_2_D3 (calcitriol) concentration and (**E**) serum calcium concentration in the male offspring at weaning (PN21) (*n* = 5). The data are expressed as mean ± SEM. Unpaired two−tailed Student’s *t*−tests or One−way ANOVA followed by Tukey’s post hoc test. * *p* < 0.05 VDD versus CT group. # *p* < 0.05 VDD + Ca versus CT group, and & *p* < 0.05 VDD + Ca versus VDD group.

To investigate the effects of perinatal VDD on the development and function of BAT, VDD was induced in female Wistar rats prior to gestation. Additionally, a separate group of rats received a VDD diet supplemented with calcium (VDD + Ca) to rule out potential effects of VDD-induced hypocalcemia on BAT physiology. As expected, serum calcidiol measurements confirmed that VDD dams became vitamin D-deficient before conception and remained deficient through weaning (Figure 1A,B). Accordingly, VDD offspring exhibited decreased serum calcidiol (Figure 1C), serum calcitriol (Figure 1D), and serum calcium concentrations (Figure 1E). The VDD + Ca rescue diet successfully induced hypovitaminosis D in dams and their offspring, while preventing a drop in calcium concentration (Figure 1C–E). These findings demonstrate that our experimental design successfully established a VDD environment during the perinatal period, negatively impacting the abundance of the active form of vitamin D [1,25(OH)_2_D_3_] in the offspring. Furthermore, the VDD + Ca diet abolished VDD-induced hypocalcemia, allowing us to isolate the effects of VDD from its impact on mineral metabolism.

### 2.2. Vit. D Deficiency Impairs Postnatal Offspring Growth and Reduces Body Fat Percentage and Lean Mass at Weaning

**Figure 2:**
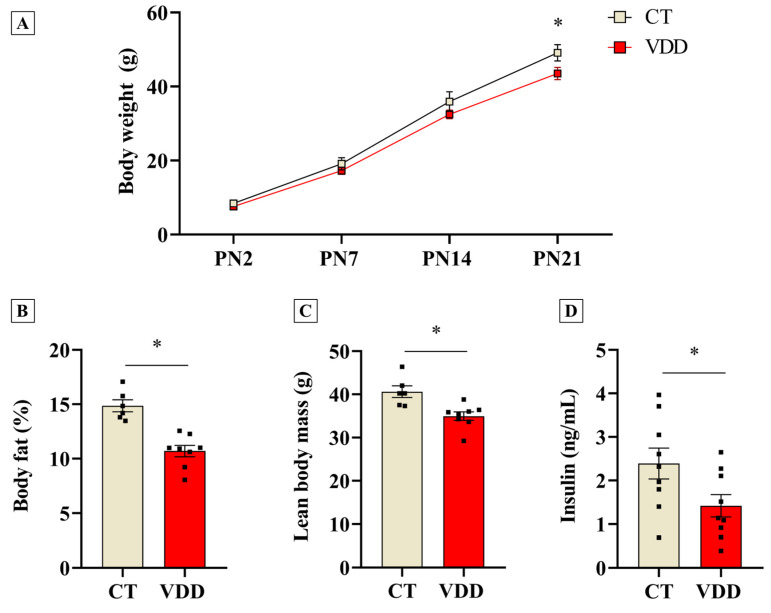
Body composition determined through magnetic resonance and metabolic parameters in the male offspring born and breastfed in Vit. D−sufficient (CT) or Vit. D−deficient (VDD) dams. (**A**) Body weight evolution in male litters during lactating period (CT *n* = 5; VDD *n* = 7). (**B**) Relative fat body mass (CT *n* = 6; VDD *n* = 8); (**C**) Absolute lean body mass (CT *n* = 6; VDD *n* = 8) and (**D**) Serum insulin concentration (*n* = 9) in the male offspring at weaning (PN21). PN: postnatal day. The data are expressed as mean ± SEM. Unpaired two−tailed Student’s *t*−tests. * *p* < 0.05 versus CT group.

Once perinatal VDD was validated, we investigated its outcomes in body composition and metabolic parameters in the offspring. The data showed that VDD male offspring had an impairment in postnatal growth, as demonstrated by reduced body weight at weaning (Figure 2A). Moreover, a lower body fat percentage and absolute lean body mass were observed in the VDD group (Figure 2B,C). Interestingly, these effects were associated with reduced serum insulin concentrations (Figure 2D), without affecting glycemia (118 ± 6 versus 125 ± 4 mg/dL in the CT group; *n* = 5). Therefore, perinatal VDD negatively affects the offspring’s growth and fat accumulation during postnatal life.

### 2.3. Perinatal Vit. D Deficiency Increases Respiratory Capacity and Induces Thermogenic Program in BAT in Newly-Weaned Rats

**Figure 3:**
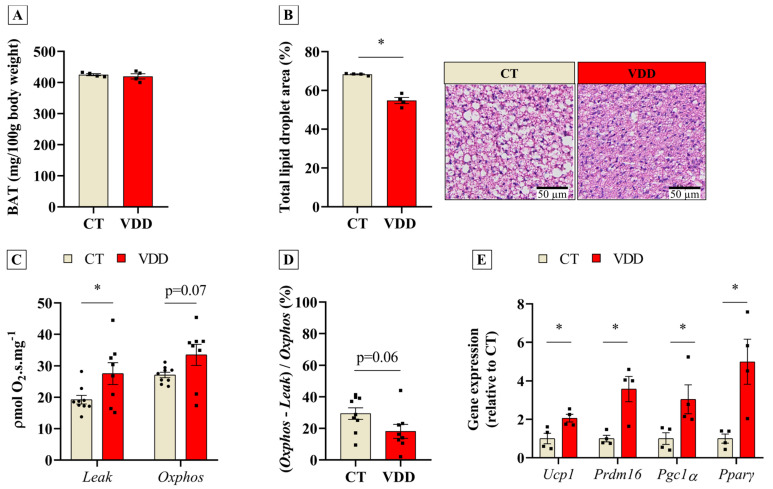
Morphological analysis, high−resolution respirometry assay, and qPCR from BAT of Vit. D−sufficient (CT) and Vit. D−deficient (VDD) male offspring at weaning (PN21). (**A**) Brown adipose tissue (BAT) relative weight (*n* = 4), (**B**) total lipid droplet area in BAT sections stained with hematoxylin−eosin (HE) (*n* = 4), (**C**) ex vivo oxygen consumption rate in BAT on leak and Oxphos respiratory states (CT *n* = 9; VDD *n* = 8), (**D**) coupling efficiency (CT *n* = 9; VDD *n* = 8), and (**E**) relative gene expression of thermogenic genes *(n* = 4) in BAT. The data are expressed as mean ± SEM. Unpaired two−tailed Student’s *t*−tests. * *p* < 0.05 versus CT group.

In order to investigate the physiological role of Vit. D in BAT morphology and function, BAT from rats exposed to perinatal VDD was studied at weaning. Although the BAT mass was not altered (Figure 3A), our histological analysis demonstrated that VDD reduced the lipid droplet area in this tissue (Figure 3B). Considering the thermogenic properties of BAT, we postulated that this finding might be related to a functional gain. High-resolution respirometry experiments revealed higher oxygen consumption in the leak state (Figure 3C), as well as a trend toward (*p* = 0.06) a lower coupled respiration in BAT biopsies from VDD rats (Figure 3D). Moreover, the thermogenic gene program (Ucp1, Prdm16, Pgc1α, and Pparγ) was upregulated in the BAT of these animals (Figure 3E) independently of norepinephrine concentration in the tissue (1101 ± 85.8 versus 1053 ± 65.8 ng/g in the CT group; *n* = 6). Altogether, these results suggest that the reduction in Vit. D signaling during embryogenesis and neonatal life stimulates BAT development and function.

### 2.4. Vit. D Deficiency Increases BAT Recruitment and Cold Tolerance in the Offspring

**Figure 4:**
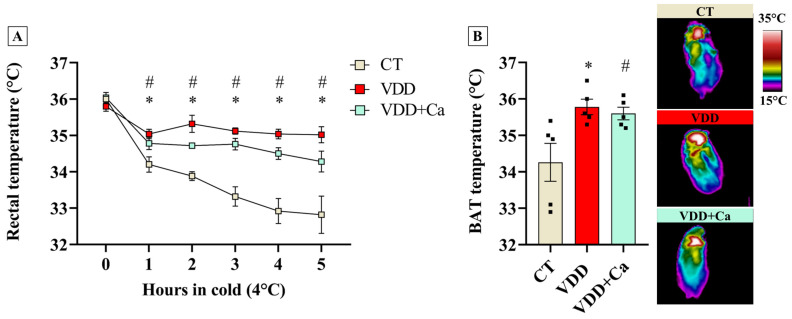
Cold tolerance test in Vit. D−sufficient (CT), Vit. D−deficient (VDD), and Vit. D−deficient supplemented with calcium (VDD + Ca) male offspring at weaning (PN21). (**A**) Rectal temperature during 5 h of cold exposure (4 °C) (*n* = 5) and (**B**) brown adipose tissue (BAT) temperature estimated through infrared thermography (*n* = 5). The pictures were taken in the last hour of cold. The data are expressed as mean ± SEM. One−way ANOVA followed by Tukey’s post hoc test. * *p* < 0.05 VDD versus CT group. # *p* < 0.05 VDD + Ca versus CT group.

To further determine whether VDD-induced BAT adaptations would have any physiological relevance in vivo, we challenged the CT and VDD offspring to cold (4 °C) for 5 h. In addition, to exclude indirect effects of VDD diet on mineral metabolism, we also used VDD + Ca rats in this experiment. As shown in Figure 4, while rectal temperature abruptly dropped during cold exposure (4 °C) in the CT group, VDD and VDD + Ca animals were resistant to cold-induced hypothermia. Interestingly, this finding was correlated with an increase in BAT temperature, suggesting that perinatal VDD, independently of hypocalcemia, enhances BAT thermogenic capacity, leading to a greater tolerance to cold challenge.

### 2.5. Recovery of Vit. D Status in Adulthood Abolishes Early BAT Adaptations

**Figure 5:**
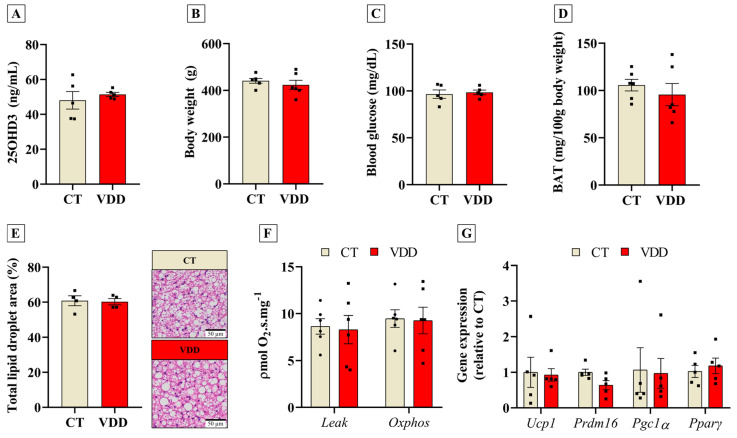
Effects of perinatal Vit. D deficiency in the adult (180 days-old) male offspring receiving a Vit. D−sufficient diet (Nuvital) after weaning. (**A**) Serum 25OHD3 (calcidiol) concentration (*n* = 5), (**B**) body weight (*n* = 6), (**C**) blood glucose (*n* = 5), (**D**) brown adipose tissue (BAT) relative mass (*n* = 6), (**E**) total lipid droplet area in BAT sections stained with HE (*n* = 4), (**F**) ex vivo oxygen consumption rate in BAT on leak and Oxphos respiratory states (*n* = 6), and (**G**) relative gene expression of thermogenic genes in BAT (*n* = 6) from the adult offspring (180−days old) born and breastfed in Vit. D−sufficient (CT) or Vit. D−deficient (VDD) mothers. The data are expressed as mean ± SEM. Unpaired two−tailed Student’s *t*−tests.

The DOHaD concept proposes that perinatal insults may induce long-term outcomes in the offspring [19]. To investigate whether VDD during gestation and lactation could permanently affect BAT, we introduced a Vit. D-sufficient diet to VDD offspring after weaning. In adult life (postnatal day 180), VDD animals receiving the Vit. D-sufficient diet had their serum calcidiol concentrations completely normalized (Figure 5A). In this context, no changes were observed in body weight, blood glucose levels, or BAT mass between the groups (Figure 5B–D). As shown in our previous work [26], adult VDD rats develop hyperinsulinemia. Unlike newly weaned VDD offspring, animals from the VDD group with Vit. D sufficiency in adult life did not show any difference in BAT lipid droplet area (Figure 5E). Moreover, BAT oxygen consumption (Figure 5F) and thermogenic−related gene expression (Figure 5G) were similar in VDD and CT offspring. Hence, Vit. D status recovery after weaning restores the BAT phenotype in VDD offspring.

## 3. Discussion

This study demonstrates that perinatal VDD enhances BAT thermogenesis and improves cold tolerance in newly weaned rats. These effects were independent of hypocalcemia and were reversed by Vit. D restoration in adulthood, suggesting that perinatal VDD exerts only transient effects on BAT physiology. Previous studies have shown that Vit. D signaling inhibits BAT thermogenic gene expression and function in vitro [45] and in transgenic mice overexpressing the human VDR under the control of an adipocyte-specific promoter (aP2) [47]. However, it remained unclear whether this non-canonical role of Vit. D played a role in physiological contexts. Our data indicate, for the first time, that VDD leads to BAT overactivation, suggesting that Vit. D nutritional status regulates BAT development during perinatal life.

The absence of calcitriol during fetal and neonatal development may enhance BAT thermogenic capacity through multiple mechanisms. While no differences in BAT mass were observed, VDD offspring exhibited reduced lipid droplet area, possibly indicating increased brown adipocyte number. Calcitriol has known antiproliferative effects, stimulating the expression of cell cycle arrest-related proteins such as p21 [48] and GADD45 [49]. Since BAT growth in rodents occurs via proliferation and differentiation of brown pre-adipocytes [50], the lack of calcitriol signaling during perinatal life may favor BAT hyperplasia. This aligns with our findings of increased expression of Prdm16 and Pparγ, both negatively regulated by calcitriol in brown adipocytes [45]. Future studies should examine whether the lack of calcitriol signaling in VDD directly stimulates BAT cell proliferation and differentiation in vivo.

Despite its generally suppressive role in cell differentiation, calcitriol can also stimulate this process in certain cell types. In C2C12 cells [51] and human myoblasts [52], calcitriol enhances myogenic gene expression and myotube formation. Interestingly, Prdm16 is known to inhibit myocytes differentiation, directing the fate of mesenchymal cells toward brown adipocytes [42]. In our study, we observed that decreased serum calcitriol levels in the weanling VDD male offspring were associated with higher Prdm16 expression in BAT and lower lean body mass. In conjunction with previous reports showing reduced soleus and EDL muscle mass in male offspring from VDD mothers [25], it is possible that the thermogenic enhancement of BAT induced by perinatal VDD may come at the expense of impaired skeletal muscle development. Although the effects of VDD on human BAT remain unknown, maternal VDD has been associated with lower muscle mass in children [53]. This finding suggests a potential mechanism by which VDD during gestation and lactation may adversely affect offspring health, possibly by influencing the commitment of common precursor cells toward the brown adipocyte lineage.

Beyond direct effects on BAT thermogenesis, alternative mechanisms may contribute. The reduction in lipid droplet area in the BAT of VDD offspring suggests altered lipid metabolism, likely involving decreased lipogenesis and/or increased lipolysis. Lipolysis is essential for thermogenic gene recruitment in response to β-adrenergic stimulation, with fatty acids enhancing Pparγ transcriptional activity, thereby promoting adipocyte differentiation and thermogenesis [54]. Notably, the histological changes in VDD animals appear linked to reduced serum insulin levels rather than BAT norepinephrine content differences. Our previous work showed that VDD male offspring exhibit impaired glucose-stimulated insulin secretion and reduced β-cell mass, independent of hypocalcemia [26], suggesting that VDD enhances BAT thermogenesis partly by diminishing insulin-mediated antilipolytic signaling in brown adipocytes.

VDD is commonly associated with hypocalcemia and secondary hyperparathyroidism. As expected, VDD offspring exhibited lower calcium levels and elevated PTH concentrations, as previously demonstrated by our group [55]. In vitro studies indicate that extracellular calcium suppresses thermogenic gene expression and impairs brown adipocyte differentiation [56], while elevated PTH can enhance BAT thermogenesis, as seen in cachexia models [57]. However, our high-calcium rescue diet experiments ruled out hypocalcemia and secondary hyperparathyroidism as primary drivers of BAT thermogenesis in VDD offspring, as normalizing calcium and PTH levels did not prevent the enhanced BAT recruitment at 4 °C or the improved cold tolerance. These findings suggest that reduced Vit. D signaling may play a direct role in promoting BAT thermogenesis. From a translational perspective, this mechanism may be relevant in clinical settings characterized by VDD where reducing energy expenditure would be beneficial, such as in chronic kidney disease [58].

In addition to the acute effects of vitamin D deficiency in enhancing BAT thermogenesis in weanling offspring, the DOHaD concept prompted us to investigate whether these effects persist into adulthood. Although metabolic programming of BAT has been previously described in a model of protein restriction [59], our data show that the BAT phenotype induced by VDD is reestablished in adulthood following normalization of vitamin D status. Previous work from our group demonstrated that maternal VDD induces compensatory mechanisms in adult offspring related to local vitamin D metabolism, including increased expression of CYP27B1 and elevated calcitriol content in skeletal muscle [25]. Furthermore, adult rats exposed to VDD showed hyperinsulinemia, which may have contributed—at least in part—to the recovery of lipid droplet area in BAT. Therefore, the normalization of the BAT phenotype in adult offspring may be associated with increased local calcitriol signaling, as BAT also expresses vitamin D-metabolizing enzymes [45], as well as with compensatory insulin signaling.

Despite the advances provided in the present study, several limitations warrant further investigation. First, the effects of perinatal VDD were demonstrated only in male offspring, and some degree of sexual dimorphism may exist, as previously observed in skeletal muscle [25] and pancreatic islets [26]. Second, since only a single adult time point was evaluated, we were unable to determine the exact time at which the enhanced BAT phenotype in VDD offspring was reversed. Third, the molecular mechanisms by which perinatal VDD increased BAT thermogenesis were not fully explored and remain to be elucidated. Finally, the calcidiol concentrations achieved through our dietary intervention reflect a condition of severe VDD, which may not be fully representative of the more common range of calcidiol levels observed in humans with VDD or vitamin D insufficiency (12–30 ng/mL).

In summary, perinatal VDD stimulates the thermogenic gene program and enhances BAT function in newly weaned male rats independently of calcium status. Moreover, we demonstrated that these effects were completely reversed in adulthood after Vit. D status recovery, indicating that calcitriol exerts a transitory inhibitory influence on BAT differentiation and function. Although BAT thermogenic function has been showed to be inversely related to obesity-related disorders in humans, its over-activation also has deleterious effects during catabolic conditions. In this way, Vit. D status may be a candidate for the management of wasting in human diseases such as kidney failure-associated cachexia.

## 4. Materials and Methods

### 4.1. Animals

**Figure 6:**
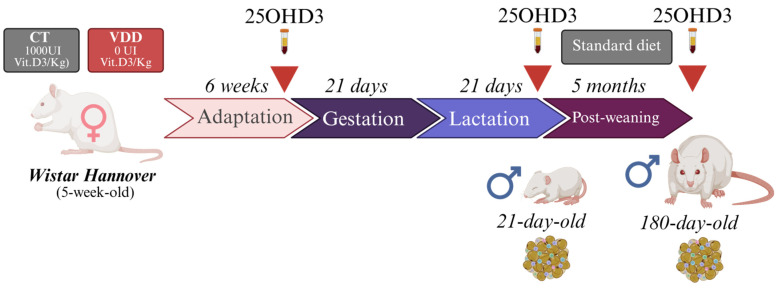
General experimental design of the study. Forty female Wistar Hannover rats were fed either a vitamin D-sufficient diet (Control group, CT; *n* = 20) or a vitamin D-deficient diet (VDD group; *n* = 20) for a 6-week adaptation period, continuing through gestation and lactation. Male offspring were evaluated either at weaning or after being maintained on a standard diet until adulthood (180 days of age). Serum 25OHD_3_ levels were measured at multiple time points in both dams and their offspring (indicated by red arrows). Brown adipose tissue (BAT) was collected at weaning and in 180−day−old male offspring for histological, respirometric, and gene expression analyses. This figure was developed by the authors in “Biorender”. https://app.biorender.com/ (accessed on 18 April 2025).

The experimental procedures were conducted according to the Brazilian College of Animal Experimentation and were approved by Ribeirão Preto Medical School of the University of São Paulo-The Ethics Committee on Animal Use (CEUA 1241/2023). Forty five−week−old female Wistar Hannover rats were randomly assigned to a fed regime with a Vit. D−sufficient diet (CT group) or a Vit. D−deficient diet (VDD group) (PragSolucões; Jaú, SP, Brazil) (Appendix A. Table A1) for 6 weeks (adaptation) and during gestation (21 days) and lactation (21 days). Pregnant rats were identified by the presence of a vaginal plug and then single-housed until parturition. On postnatal day 2 (PN2), the litter size was adjusted to eight pups (4 males and 4 females) per dam and their body weight was monitored once a week. At weaning (PN21), the male offspring were submitted to body composition analysis, the cold tolerance test, or anesthetized with isoflurane and euthanized through decapitation for blood and BAT collection (Figure 6). A separated group of dams were fed with a vitamin D−deficient diet supplemented with (VDD + Ca) to exclude the effects of hypocalcemia on BAT thermogenic function in vivo. To investigate long-term effects of perinatal VDD in the offspring, a group of rats received a Nuvilab standard diet (Nuvital, Quimta) after weaning until adulthood (180 days of life). The animals used in the experiments were housed in a facility with a 12 h light–dark cycle, at room temperature (22 °C) with free access to food and water. At least one researcher was aware of the group allocation during the experiments.

### 4.2. Body Composition Analysis

The newly weaned rats were restrained for 60 s for the body lean mass and body fat mass assessment through whole-body analyzer (Bruker’s minispec Whole Body Composition Analyzer, Billerica, MA, USA) based on TD-NMR.

### 4.3. Cold Tolerance Test

The male offspring were weaned at postnatal day 21 (PN21) at zeitgeber time 0 (ZT0) and briefly restrained for the interescapular shaving (2 cm^2^). Afterward, the animals were allocated in individual cages containing a thin layer of bedding, without food and with free access to water. One hour after shaving (ZT1), the rats were transferred to a cold-room (4 °C) for 5 h. The rectal temperature (BAT-10 thermometer, Physitemp, Clifton, NJ, USA) and surface interescapular temperature (Infrared Thermography; FLIR instruments, Wilsonville, OR, USA) were recorded throughout the cold challenge. For rectal temperature measurements, RET-3 probes (Physitemp, Clifton, NJ, USA) were pre-coated with petroleum jelly to ensure animal welfare and minimize stress. Interescapular temperature values were obtained by identifying the hottest surface point using the free-access FLIR Tolls software (Version 6.4).

### 4.4. Tissue and Blood Collection

After the euthanasia, 1 mL of the collected blood was kept in ice and centrifuged (1500× *g*; 15 min; 4 °C) for serum collection. The serums were stored in −20 °C until the hormones and metabolites analysis. The interescapular brown adipose tissue was harvested and separated from adjacent muscular and white fat tissue in ice-cold NaCl (0.9%). After that, the tissue was frozen in liquid nitrogen and stored in −80 °C or fixed in paraformaldehyde 4% for histological analysis.

### 4.5. Hormonal and Metabolites Measurements

The serum calcidiol (25OHD3) and calcitriol (1,25[OH]2D3) quantifications were made using a chemiluminescence analyser (DiaSorin, Liaizon^®^ XL, Austin, TX, USA) and for calcium determination, an Agilent 55B AA Atomic Absorption Spectrometer (Agilent Technologies, Santa Clara, CA, USA) was used. Blood glucose was obtained in the tail blood drip throughout a commercial glucometer (Accu-Chek Performa, Roche Applied Science, São Paulo, Brazil). Serum insulin levels were measured using an ELISA kit (Merck Millipore, Burlington, MA, USA) according to the manufacturer’s instructions.

### 4.6. High-Resolution Respirometry

The oxygen consumption from BAT biopsies was measured using an Oroboros O2K oxygraph (Bioblast, Innsbruck, Tirol, Austria) following the method proposed by Dechandt et al. [60]. For this, the upper right lobe of BAT was sectioned and cut in 1 mm^3^ pieces in a cold BIOPS solution (CaK2EGTA [2.77 mM]; K2EGTA [7.23 mM]; free calcium [0.1 M], imidazole [20 mM], taurine [20 mM], 2-(N-Morpholino)ethanesulfonic acid potassium salt [20 mM], dithiothreitol [0.5 mM]; MgCl2.6H2O [6.56 mM]; phosphocreatine [15 mM]; ATP [5.77 mM]; pH = 7.1]). The fragments were washed under agitation in BIOPS solution plus 0.1% BSA for 20 min, and then permeabilized in BIOPS solutions containing sapoponin (5 µg/mL) for 20 min. The fragments were dried on filter paper, and approximately 8 mg of tissue was transferred in duplicate to the oxygraph chamber containing 2 mL of MIR05 solution (sucrose [110 mM], potassium lactobionate [60 mM]; EGTA [0.5 mM]; MgCl2.6H2O [3 mM]; taurine [20 mM]; KH2PO4 [10 mM]; HEPES [20 mM]; BSA [1 g/L]; pH 7.1, 37 °C). Uncoupled (Leak) and phosphorylative (Oxphos) respiratory states were obtained, respectively, by quantifying the average oxygen consumption rate after the addition of 20 µL of succinate [20 mM] and 8 µL of ADP [0.5 mM], subtracted from the average oxygen consumption rate after the addition of 10 µL of NaCN [1 M]. The coupled respiration coupling index was calculated from the ratio between the difference of Oxphos and Leak by Oxphos [(Oxphos − Leak)/Oxphos]. All data obtained were normalized by the weight of the BAT used in the respirometric analysis.

### 4.7. Histological Analysis

Small BAT fragments (0.125 cm^3^) were fixed in 4% paraformaldehyde for 48 h. Subsequently, the tissue was dehydrated with increasing ethanol concentration and then cleared in xylol. BAT was embedded in paraffin and sectioned into 6 µm slices using a microtome (Rotating Microtome Leica RM200, Leica ByoSystems, Wetzlar, Germany). The slices were stained with HE, and images acquired using a ScanScope system Olympus BX61VS (Olympus Corporation, Hachioji, Japan) with a 40× objective lens. The mean lipid droplet area was calculated using ImageJ software (Fiji is Just, Version 1.54f; National Institutes of Health, Bethesda, MD, USA) through the threshold method.

### 4.8. Norepinephrine Measurements

The method for measuring BAT norepinephrine was adapted from Garófalo et al. [61]. Briefly, 50 mg of BAT was homogenized in 1.8 mL of buffer containing HClO_4_ [0.2 N], EDTA [1 mM], and sodium metabisulphite [1%]. Afterward, the homogenate was centrifuged (5000× *g*, 10 min, 4 °C) and 1.6 mL of the supernatant was transferred to tubes containing Tris [2 M] (pH 8.9), sodium metabisulphite [0.5%], EDTA [2.5%], and 50 mg of activated alumina. Each sample was added with 20 μL of 3,4-Dihydroxybenzylamine as internal standard. The samples were shaken for 20 min and the supernatant was then discarded. Alumina was washed three times with 1.5 mL washer solution (Tris [5 mM]), sodium metabisulphite [0.02%], EDTA [0.1 mM]), and then dried under vacuum for 5 min for elution in 800 µL of HClO_4_ [0.1 N] under agitation. The solution was centrifuged (5000× *g* 5 min 4 °C) and the supernatant was collected for norepinephrine measurements through high-performance liquid chromatography (Shimadzu, Milton Keynes, UK).

### 4.9. Quantitative PCR

Total RNA from BAT was extracted through TRI-Reagent (Sigma-Aldrich, Darmstadt, Germany) and quantified by spectrophotometry (NanoDropOne; Thermo Scientific, Waltham, MA, USA). One μg of purified RNA was reverse transcribed using SuperScript IV First-Strand Synthesis System (Invitrogen, Carlsbad, CA, USA). qPCR was performed using PowerUp™ SYBR™ Green Master Mix (Applied Biosystems, Foster City, CA, USA) and specific primers (Table 1). The reaction was followed by a dissociation curve analysis, and the relative gene expression was calculated as proposed by Pfaffl et.al [62] using Rpl39 as a housekeeping gene.

### 4.10. Statistics

The data were presented as mean ± standard error of the mean. Normality was assessed through Shapiro Wilk test and comparison between the groups was made using unpaired two-tailed t-Student test or One-way ANOVA followed by Tukey’s post hoc test. Statistical significance was set at *p* < 0.05. Outliers were identified and excluded using ROUT test (Q = 1%). Each pup was considered an experimental unit and animals were obtained from a minimum of two different litters, unless otherwise stated.

## Figures and Tables

**Table 1:** Primer sequences used in qPCR.

Target Gene	Forward (5′–3′)	Reverse (5′–3′)
*Ucp1*	CCGGTGGATGTGGTAAAAAC	GTTTTTACCACATCCACCGG
*Prdm16*	CAGCACGGTGAAGCCATTC	GCGTGCATCCGCTTTG
*Pgc1α*	GCTTGACTGGCGTCATTCA	ACAGAGTCTTGGCTGCACATGT
*Pparγ*	GTGCCAGTTTCGATCCGTAGA	GGCCAGCATCGTGTAGATGA
*Rpl39*	TCCTGGCAAAGAAACAAAAGC	TAGACCCAGCTTCGTTCTCCT

## Data Availability

The original contributions presented in this study are included in the article; further inquires can be directed to the corresponding author.

## Appendix A

**Table A1:** Composition of the Vit. D-sufficient (CT), Vit. D-deficient diet (VDD), and Vit. D-deficient diet supplemented with calcium (Ca^2+^) (VDD + Ca).

Nutrient (g/kg)	AIN93G (CT)	AIN93G Without Vit. D (VDD)	AIN93G Without Vit. D + Ca^2+^ (VDD + Ca)
Corn starch	397.50	397.50	173
Casein	200.00	200.00	200.00
Starch dextrinated	132.00	132.00	100
Sucrose	100.00	100.00	100.00
Soy bean oil	70	70	70
L-Cystine	3.00	3.00	3.00
Choline Bitartrate	2.50	2.50	2.50
Butylated Hydroxytoluene	0.014	0.014	0.014
Mineral mix	35.00	35.00	35.00
Vitamin mix	10.00	10.00	10.00
Fibre	50.00	50.00	50.00
Vit. D3	25 × 10^−6^	0.00	0.00
Calcium carbonate	0	0	35.00
Sodium phosphate dibasic anhydrous	0	0	21.50
Lactose	0	0	200.00
